# Necrotizing Skin and Soft Tissue Infection after Gluteal Augmentation in a Perioperatively Asymptomatic COVID-19 Patient—Complications of the Post-Lockdown Era? A Case Report

**DOI:** 10.3390/medicina59050914

**Published:** 2023-05-10

**Authors:** Milan Stojičić, Milana Jurišić, Milana Marinković, Milan Jovanović, Aleksa Igić, Maja Nikolić Živanović

**Affiliations:** 1 Clinic for Burns, Plastic and Reconstructive Surgery, University Clinical Center of Serbia, 11000 Belgrade, Serbia; 2 Faculty of Medicine, University of Belgrade, 11000 Belgrade, Serbia; 3 Center for Radiology and Magnetic Resonance Imaging, Department of Interventional Radiology, University Clinical Center of Serbia, 11000 Belgrade, Serbia

**Keywords:** gluteal augmentation, necrotizing skin and soft tissue infection, COVID-19, aesthetic surgery, necrotizing fasciitis

## Abstract

*Introduction*: Aesthetic surgery procedures are generally done in a relatively healthy population and carry a rather low risk compared to other surgical specialties. The incidence of complications in aesthetic surgery varies greatly depending on the type, wound cleanliness regarding the anatomical site, complexity of the surgery, patient’s age, and comorbidities but is generally considered low. The overall incidence of surgical site infections (SSIs) in all aesthetic surgical procedures is around 1% in most of the literature while cases of necrotizing soft tissue infections are mostly found as individual reports. In contrast, treating COVID-19 patients is still challenging with many diverse outcomes. Surgical stress and general anesthesia are known mediators of cellular immunity impairment while studies regarding COVID-19 infection unquestionably have shown the deterioration of adaptive immunity by SARS-CoV-2. Adding COVID-19 to the modern surgical equation raises the question of immunocompetence in surgical patients. The main question of the modern post-lockdown world is: what could be expected in the postoperative period of perioperatively asymptomatic COVID-19 patients after aesthetic surgery? *Case report*: Here, we present a purulent, complicated, necrotizing skin and soft tissue infection (NSTI) after gluteal augmentation most likely triggered by SARS-CoV-2-induced immunosuppression followed by progressive COVID-19 pneumonia in an otherwise healthy, young patient. To the best of our knowledge, this is the first report of such adverse events in aesthetic surgery related to COVID-19. *Conclusion*: Aesthetic surgery in patients during the incubation period of COVID-19 or in asymptomatic patients could pose a significant risk for surgical complications, including severe systemic infections and implant loss as well as severe pulmonary and other COVID-19-associated complications.

## 1. Introduction

Since the first case of COVID-19 was reported in December 2019, there have been significant changes in the organization of the healthcare system worldwide [1]. During the COVID-19 pandemic, the majority of countries suspended most elective surgery as a protective measure against SARS-CoV-2 transmission and an alleviation measure on the healthcare system, decreasing the rate of surgical aesthetic procedures. On the flip side, increased usage of video conferencing and social media during lockdown seems to have led to a rise in demand for cosmetic surgical and non-surgical procedures post-lockdown [2]. Nonetheless, the COVID-19 pandemic has changed healthcare immensely, bringing a new array of perioperative and postoperative risk factors as well as complications.

Aesthetic surgery procedures are generally done in a relatively healthy population and carry a rather low risk compared to other surgical specialties. The incidence of complications in aesthetic surgery varies greatly depending on the type, wound cleanliness regarding the anatomical site, complexity of the surgery, patient’s age, and comorbidities but is generally considered low. The overall incidence of surgical site infections (SSIs) in all aesthetic surgical procedures is around 1% in most of the literature [3,4,5]. For example, a study regarding breast surgery found the incidence of SSIs to be 1.4–3.2% [6]. Given the anatomical site being proximate to the anus, the incidence of infections following buttock augmentation is expectedly higher, and complications can be more severe. Bruner et al. reported the infection rate of 13.3%, further managing to decrease these complications to 2% after introducing strict prophylactic preoperative and postoperative protocols [7]. These incidences were related to gluteal augmentation with fat transfer. Infection rates after gluteal augmentation with solid silicone implants, however, are reported in the literature from 1% to 7%. The most common pathogen found in a systematic review by Shah et al. was Staphylococcus aureus, while Escherichia coli was found in only one patient [8].

Treating COVID-19 patients is still challenging with many diverse outcomes. Some studies suggest about 20% of hospitalized COVID-19 patients require intensive care, and more than 75% of them require oxygen therapy [9,10]. Occasionally, a potentially severe life-threatening multisystem inflammatory syndrome (MIS) can appear in the period of 2–6 weeks after the first symptoms of COVID-19 [11].

Necrotizing fasciitis (NF), a rare yet life-threatening necrotizing skin and soft tissue infection with a prevalence from 0.40 cases per 100,000 to 15.5 cases per 100,000 population, was found to be most commonly associated with surgery, immunosuppression, and diabetes [12,13]. The main question of the modern post-lockdown world is: what could be expected in the postoperative period of perioperatively asymptomatic COVID-19 patients after aesthetic surgery? Here, we present necrotizing fasciitis after gluteal augmentation, most likely triggered by SARS-CoV-2-induced immunosuppression followed by progressive COVID-19 pneumonia in an otherwise healthy, young patient. To the best of our knowledge, this is the first report of such adverse events in aesthetic surgery related to COVID-19.

## 2. Case Report

A 39-year-old, non-vaccinated, female patient was admitted to COVID Hospital due to a high fever, weakness, and mild respiratory symptoms followed by a positive RNA test for SARS-CoV-2 infection. On admission, the patient reported she had gluteal augmentation with solid silicone implants (manufacturer Impalntech Associates, Inc., Ventura, CA, USA, REF CCB5-0) 13 days prior and was now complaining of pain in the gluteal region. The patient received antibiotics (Amoxicillin/Clavulanic acid) during the 7 days postoperatively, as noted in her medical history. Additionally, insulin resistance was reported by the patient with no other chronic diseases, as well as prior breast augmentation with silicone implants.

**Figure 1:**
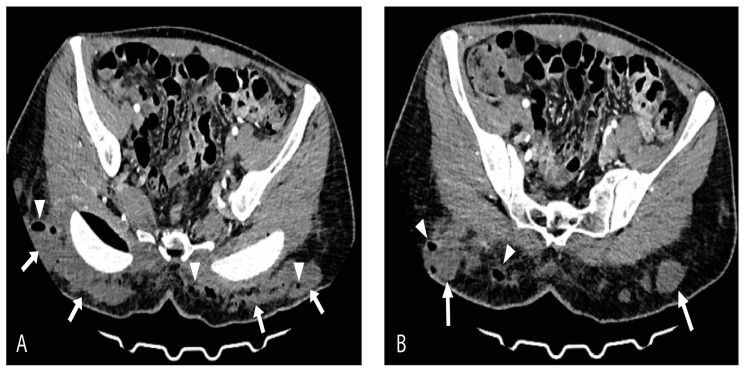
Axial-contrast-enhanced CT scan views (**A**) and (**B**) show massive multifocal and confluent nonorganized fluid collections (arrows) with foci of gas (arrowheads) and surrounding fat stranding within subcutaneous adipose tissue of gluteal region bilaterally. Posterior parts of dissected major gluteal muscles are also involved.

On the day of admission, the patient presented with a high fever (up to 40 °C), tachycardia, tachypnea, and dry cough. Pulse oximetry showed normal oxygen saturation with no need for supplemental oxygen. Laboratory results revealed lymphopenia (1.23 × 10^9^/L), anemia (hemoglobin 112 g/L and red blood cells 3.57 × 10^12^/L), elevated fibrinogen (7.2 g/L), D-dimer (2.07 g/L), Interleukin-6 (20 pg/mL), lactate dehydrogenase (LDH 781 U/L), ferritin (387 U/L), creatine kinase (CK 212 U/L), and C-reactive protein (CRP up to 253 mg/L) while the neutrophil-to-lymphocyte ratio (NLR) was 3.9. Chest radiography showed normal findings. On physical examination, a dehisced wound with an abundant purulent secretion in the gluteal augmentation surgical site was noted as well as dusky, livid discolored gluteal skin. Additionally, palpation revealed the presence of crepitations in the gluteal region bilaterally. A computed tomography (CT) scan revealed multiple focal and confluent nonorganized fluid collections with foci of gas, fat stranding within the subcutaneous fat of the gluteal region posterior to the gluteal implants, and the posterior parts of dissected major gluteal muscles (Figure 1A,B).

**Figure 2:**
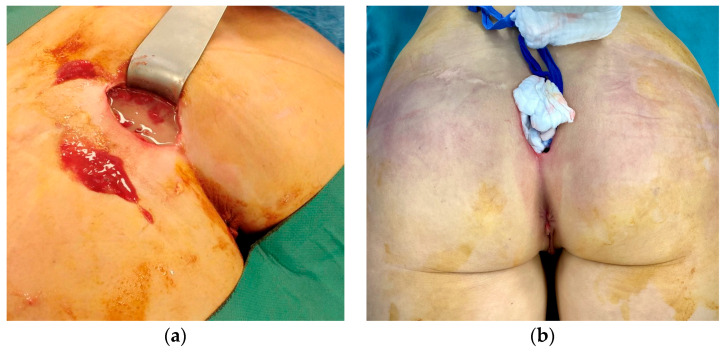
The intraoperative findings of well-positioned intramuscular implants surrounded by a large amount of pus (**a**) and dusky, livid discoloration of gluteal skin (**b**).

**Figure 3:**
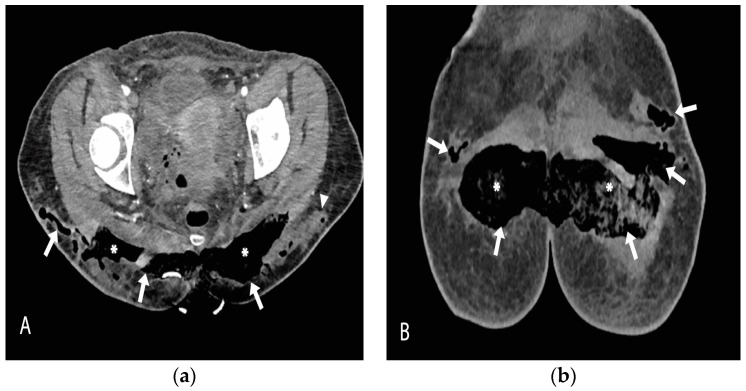
Contrast-enhanced CT scan axial (**a**) and coronal (**b**) views, obtained after the removal of gluteal implants, reveal almost completely drained fluid collections, which have been replaced by air (arrows). The intragluteal space is packed with gauze (asterisk). There are some residual focal fluid collections in the very lateral part of the left gluteal region (arrowhead).

The patient was urgently taken to the operating room for the removal of the gluteal implants and surgical debridement (Figure 2). The intraoperative findings of well-positioned intramuscular implants surrounded by large quantities of pus correlated with the CT scan and local status confirmed the diagnosis of necrotizing fasciitis. A high dose of empiric antibiotics (Meropenem, Vancomycin, and Metronidazole) was administered. *Escherichia coli* spp. was isolated from the wound swab and pus sample, and the antimicrobial therapy was corrected as per the antibiogram. Anaerobe cultures were negative. SARS-CoV-2 infection was treated according to the current National COVID-19 Treatment Protocol. Antiviral therapy (Remdesivir) was administered. A control CT scan of the surgical site infection was done after 3 days, shown in Figure 3, and the patient was placed in the intensive care unit (ICU) for extensive monitoring.

**Figure 4:**
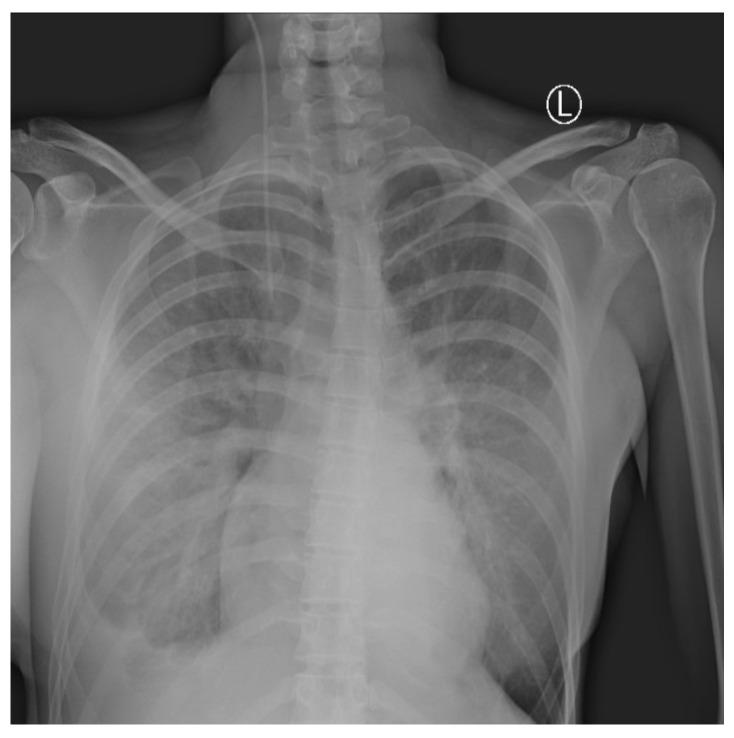
Chest X-ray shows right-sided pleural effusion with bilateral lung interstitial thickening. The interpretation of the image was partially limited due to superposition of breast implants.

Daily surgical debridement and drainage were performed, and control of the infection was obtained. Regardless of the improvement of the NSTI, in the following days, the respiratory symptoms worsened in terms of dyspnea, with peripheral blood oxygen level measured at 88%. Oxygen therapy was administered with maximum oxygen flow up to 12 L/min, leading to control of oxygen saturation of 96%. A chest radiography revealed signs of progressive bilateral pneumonia with a right-sided pleural effusion (Figure 4). High fever with high-inflammation blood markers persisted hence the antimicrobial therapy was corrected. Tigecycline, Metronidazole, and Fluconazole were administered.

**Figure 5:**
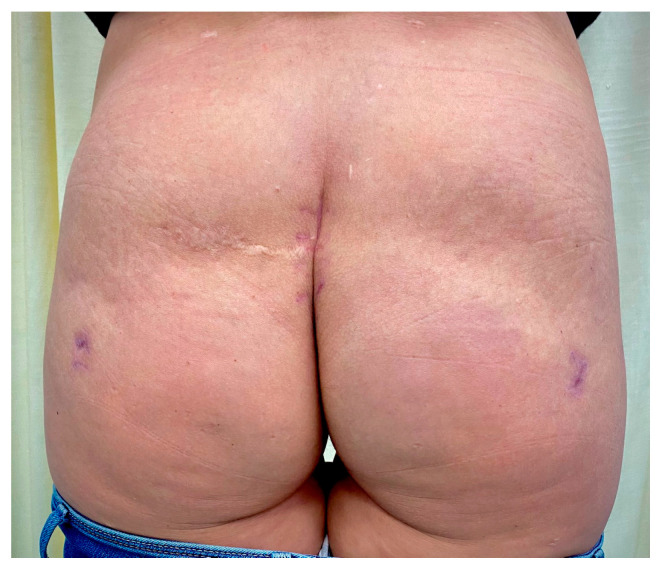
The findings after 8 weeks showed no scar abnormalities, while a contour deformity at the implantation site was present.

A good therapeutic response was obtained to the given therapy. After stabilization of respiratory symptoms and obtaining negative wound cultures, the wound was sutured, and active drains were placed. The postoperative period passed without further complications. The drains were removed on the 7th postoperative day after suturing, and the patient left the hospital after 14 days of hospitalization. The patient spent a total of 11 days in the ICU and 3 days in the semi-intensive care unit (SI-CU). On follow-up after 8 weeks, the scar showed no abnormalities; however, there was a contour deformity at the implantation site (Figure 5).

## 3. Discussion

Given the proximity of the anus to the surgical incision, SSIs after gluteal augmentation in the literature are found to be expectedly higher. In a study of 200 patients, Senderoff et al. found the infection rate to be 6.5% for both subfascially and intramuscularly placed implants, while the implant infection rate was 3.8% [14]. Another systematic review assessing the safety and efficacy of gluteal augmentation found infection rates after solid implant gluteal augmentation to be 1.94%, with implant revision rates of 1.68% and implant removal rates of 0.72%. Risk factors commonly found for SSIs in plastic surgery include older age, female gender, elevated body mass index (BMI), tobacco smoking, diabetes mellitus, and most importantly, an immunocompromised status [3,15]. Our patient, being a nonsmoking, young, otherwise healthy woman, had no common risk factors for SSIs after gluteal augmentation or for the more severe NSTI commonly associated with the immunocompromised and the elderly. No complications were reported by the patient regarding her previous breast augmentation surgery. Severe complications such as life-threatening NSTIs after gluteal augmentation have been described in the literature only as individual reports. One patient had gluteal augmentation with fat transfer, while two of the three reported patients found had gluteal augmentation with solid silicone implants. In one patient, pregnancy was found as the cause of immunosuppression; the other patient’s medical history was undisclosed [16,17,18].

Some of the most common pathogens in SSIs reported are *Staphylococcus aureus*, *Staphylococcus epidermidis*, *group A Streptococcus*, and *Pseudomonas aeruginosa* [19,20]. *Escherichia coli* is the third most frequently isolated bacteria, though it is rarely a monomicrobial source of complicated necrotizing soft tissue infections [21,22]. The type of bacteria to colonize the wound mostly depends on the surgical site localization. Surgical incisions in proximity to the intestinal tract or female genitalia are more likely to have a mixed gram-positive and gram-negative flora with both facultative and anaerobic bacteria, usually responsible for complicated, purulent NSTIs. Management of SSIs is outlined in great detail in a guideline by Stevens et al. that notes that improper and untimely management can evolve into complicated, necrotizing skin and soft tissue infection. These infections are especially found to be related to surgical and immunocompromised patients, such as patients with autoimmune diseases or patients receiving immunomodulatory therapy [22]. CT findings of bilateral fluid collections filled with gas in subcutaneous adipose tissue, along with fat stranding and involvement of gluteus major muscles, with a rapid progression of systemic symptoms in our patient, presented a worrisome sign of a rather complicated infection. Given the anatomical region, the suspicion of polymicrobial necrotizing fasciitis was high, and the patient’s swab samples and pus samples were tested for aerobes and anaerobes. A fairly rare result of monomicrobial *Escherichia coli* necrotizing infection was obtained, given the anaerobe cultures were negative. Though not as common, various reports of virulent forms of *Escherichia coli* spp. as a single cause of complicated NSTI, such as necrotizing fasciitis, have been emerging in the literature. In most reports, these infections seem to be related to the immunocompetence of the patients rather than to the virulence of the bacteria [23,24].

Surgery is found to modify the immunologic profile of patients by the transition of the lymphocytes from the vascular bed to the lymphatic system and subsequent postoperative lymphopenia. In addition, the ischemia/reperfusion injury during surgery is found to cause the release of both pro- and anti-inflammatory cytokines, mostly IL6, IL8, IL10, and TNFα, causing a neutrophilic immunologic response [25,26]. Thus, surgical stress and general anesthesia are known mediators of cellular immunity impairment [27]. Moreover, COVID-19 shows a specific immune profile in infected patients. One large study from Wuhan, China showed that lymphopenia was present in 83.2% of the patients on admission [25]. Additional studies showed a reduction in both T helper and suppressor T cells in COVID-19 patients, clearly outlining the deterioration of adaptive immunity [28]. Adding COVID-19 to the modern surgical equation raised the question of immunocompetence in surgical patients. A small matched cohort study by Doglietto et al. found COVID-19 to be an added risk factor for increased postoperative morbidity and mortality in SARS-CoV-2-positive patients as well as in preoperatively negative patients who tested positive in the week following surgery [29]. However, there are no available data on the impact of COVID-19 on early postoperative morbidity in patients after gluteal augmentation with solid silicone implants. In our patient, COVID-19 symptoms developed concomitantly with symptoms of the surgical site infection on the 13th postoperative day. In the post-lockdown era, the most important question concerns potentially asymptomatic patients and patients in the incubation period scheduled for elective surgery.

A study assessing the outcomes of patients undergoing elective surgery in the incubation period reported mortality rates of 20.6%, ICU admission requirement for 44.1% of patients, and a 100% rate of CT-verified pneumonia. In the study, 58.8% of patients had 1 or more comorbidities, and the most common complications described were ARDS, shock, secondary infection, cardiological complications, and acute kidney injury [30]. Another study found that patients who tested negative prior to elective surgery and then tested positive in the following 30 days postoperatively had a higher incidence of major, pulmonary, ischemic, or any complications compared to patients who tested negative before and after surgery (32.9 vs. 9.3%, 23.0 vs. 2.3%, 4.2 vs. 1.9%, and 41.0 vs. 13.5%, respectively) and were hospitalized longer. Major complications included the need for readmission, reoperation, or lethal outcome [31]. An international, multicenter, cohort study of 235 hospitals from 24 countries found pulmonary complications associated with high 30-day mortality rates in elective patients with a postoperative COVID-19 diagnosis in almost 1/3 of the patients [32]. In addition to COVID-19 presenting a major risk factor for surgical complications, surgery-induced cellular immunity impairment presents a major risk factor for a more severe form of COVID-19 pneumonia in operated patients. In our patient, despite the prompt and regular surgical debridement of the NSTI and the administered antimicrobial therapy, high fever and elevated inflammation parameters still persisted followed by a sudden drop in oxygen saturation. Persisting bilateral pneumonia complicated by a pleural effusion was later radiographically confirmed.

As aforementioned, the immunologic changes following surgery promote lymphopenia accompanied by a neutrophilic immunologic response, increasing the neutrophil-to-lymphocyte ratio (NLR). The NLR is an indicator of the balance between inflammation represented by the neutrophil count, and adaptive immunity is represented by the lymphocyte count [33]. The high NLR in COVID-19 patients was found to be linked to higher mortality, a more severe form of pneumonia, and an increased need for mechanical ventilation [34]. Nevertheless, one large study showed the NLR to be an important preoperative indicator for 1-year mortality in plastic and reconstructive surgery patients, emphasizing the importance of careful evaluation of laboratory parameters and PCR testing prior to surgery [35]. The preoperative NLR status in our patient is unknown, giving us no predictive value, but could be helpful in depicting the immunosuppressive role of COVID-19 given the simultaneous beginning of both COVID-19 and surgical complications following the laboratory-verified increased NLR. It seemed likely that the immunocompromised status of our patient was initially caused by surgery and exacerbated by the COVID-19. The synergistic effect of the two events eventually led to the severe necrotizing infection caused by *E. coli* as a monomicrobial source.

Several survey-based articles express the concern of plastic surgeons regarding the resumption of day-to-day aesthetic surgery [36,37,38]. In addition to COVID-19 posing a potential risk for NSTI or loss of implants, surgery during COVID-19 increases the risk for severe pneumonia, requiring admission to the ICU and, in extreme cases, mechanical ventilation, a nightmare scenario for both aesthetic surgeons and patients. Accurate reporting of such adverse events, even in individual cases, is currently of utmost importance. Further research on larger aesthetic surgery patient samples is necessary. Encouraging results of safe resumption of aesthetic surgery are coming to light, provided all safety and screening methods are employed [39,40].

## 4. Conclusions

Complications such as surgical site infections after gluteal augmentation are well known to most experienced aesthetic surgeons. Nonetheless, the altered immune state during COVID-19 infection could increase the incidence and severity of these complications. Aesthetic surgery in patients during the incubation period of COVID-19 or in asymptomatic patients could pose a significant risk for surgical complications, including necrotizing skin and soft tissue infections, severe systemic infections, implant loss, and severe pulmonary and other COVID-19-associated complications. PCR testing prior to surgery should be mandatory. Detailed anamnestic data regarding the time of previous COVID-19 infection, severity, and complications should be obtained. In the first 30 days of the postoperative period, follow-up should be frequent in more complex surgical cases. Further investigation into the topic and gathering of a more significant body of data are necessary.

## Data Availability

No available data.
